# The Impact of Biomarkers in Pancreatic Ductal Adenocarcinoma on Diagnosis, Surveillance and Therapy

**DOI:** 10.3390/cancers14010217

**Published:** 2022-01-03

**Authors:** Niklas Sturm, Thomas J. Ettrich, Lukas Perkhofer

**Affiliations:** Department of Internal Medicine I, University Hospital Ulm, Albert-Einstein-Allee 23, 89081 Ulm, Germany; niklas.sturm@uniklinik-ulm.de (N.S.); thomas.ettrich@uniklinik-ulm.de (T.J.E.)

**Keywords:** pancreatic ductal adenocarcinoma, biomarker, targeted therapy, molecular diagnostics

## Abstract

**Simple Summary:**

Pancreatic ductal adenocarcinoma is a leading cause of cancer death worldwide. Due to the frequently late diagnosis, early metastasis and high therapy resistance curation is rare and prognosis remains poor overall. To provide early diagnostic and therapeutic predictors, various molecules from blood, tissue and other origin e.g., saliva, urine and stool, have been identified as biomarkers. This review summarizes current trends in biomarkers for diagnosis and therapy of pancreatic ductal adenocarcinoma.

**Abstract:**

Pancreatic ductal adenocarcinoma (PDAC) is still difficult to treat due to insufficient methods for early diagnosis and prediction of therapy response. Furthermore, surveillance after curatively intended surgery lacks adequate methods for timely detection of recurrence. Therefore, several molecules have been analyzed as predictors of recurrence or early detection of PDAC. Enhanced understanding of molecular tumorigenesis and treatment response triggered the identification of novel biomarkers as predictors for response to conventional chemotherapy or targeted therapy. In conclusion, progress has been made especially in the prediction of therapy response with biomarkers. The use of molecules for early detection and recurrence of PDAC is still at an early stage, but there are promising approaches in noninvasive biomarkers, composite panels and scores that can already ameliorate the current clinical practice. The present review summarizes the current state of research on biomarkers for diagnosis and therapy of pancreatic cancer.

## 1. Introduction

According to current epidemiological studies, pancreatic ductal adenocarcinoma (PDAC) is the 14th most common cancer type worldwide and the 7th leading cause of cancer death [1,2]. The incidence rises with a greater emphasis on Europe and North America amongst others due to risk factors associated with higher economic status [1,3], including tobacco smoking [4], heavy alcohol consumption [4,5], obesity and high BMI [4,6], diabetes mellitus [7], total serum cholesterol [8] and reduced physical activity [4].

Due to a lack of adequate screening methods and late onset of symptoms in early stages, the prognosis for pancreatic cancer is still poor. At initial diagnosis, less than 20% of all patients have a resectable disease, and accordingly the overall five-year survival rate among patients with pancreatic cancer is <5% (16). Furthermore, up to 76.6% of patients who underwent curative-intended surgical treatment develop early disease recurrence with a median relapse free survival (RFS) of 11.7 months [9] and a five- year survival rate of 12–27% [10].

Conventional surveillance approaches are incapable to significantly improve postoperative survival [10,11]. Therefore, establishing biomarker-assisted surveillance has the potential to predict early recurrence.

Diagnosis of PDAC is further hampered, as it commonly arises out of precusor lesions that are usually not accessible to conventional diagnostics. Precursor lesions are namely Acinar to ductal metaplasia (ADM) and pancreatic intraepithelial neoplasias (PanINs) which accumulate genetic alterations that ultimately lead to the development of an invasive malignancy [12,13,14,15]. Intraductal papillary mucinous neoplasms (IPMNs) represent precursor lesions with a special status, as they are accessible to conventional imaging techniques and therefore can be risk classified based on imaging criteria [16]. However, having an acceptable sensitivity of 83% in a pooled meta-analysis, these criteria lack adequate specificity [17]. Furthermore, up to 11% of radiographic low-risk classified pancreatic lesions that underwent surgery were afterwards histologically confirmed as malignant lesions [17]. On the other hand, the risk of surgical overtherapy in patients with IPMN or mucinous cystadenomas (MCN) is high, and guidelines for the management of precursor lesions of pancreatic cancer are inconsistent [18]. Therefore, the development of new non-invasive biomarkers is crucial to improve early diagnosis and surveillance of precursor lesions of PDAC to aid surgical decision making.

Treatment of advanced stage PDAC includes various chemotherapy regimens. Even with the most effective protocol the FOLFIRINOX (5-FU, Leucovorin, Irinotecan and Oxaliplatin) regimen median overall survival does not surpass 11.1 months [19]. Furthermore, calculation of patient-related toxicity has gained importance due to the establishment of new therapies and the intention to maintain a high quality of life during systemic therapy. Therefore, biomarkers to predict treatment response and toxicity for targeted therapy and classical cytoreductive agents are essential to individualize treatment.

Due to the high importance of new biomarkers for early diagnosis of occurrence and recurrence of PDAC as well as treatment response in advanced-stage tumor disease, this has been a field of interest over the past years. This review aims to outline the current state of research and the implementation of new biomarkers in clinical practice.

## 2. The Role of Biomarkers in Diagnosis of Pancreatic Ductal Adenocarcinoma

Due to limited therapeutic options in advanced PDAC, early detection is even more important for prognosis. The development of diagnostic biomarkers is a promising step for screening high-risk populations.

### 2.1. CA19-9, CEA and Other Carbohydrate Antigens

Cell surface carbohydrate structures are associated with different roles in intercellular communication, structural and protective characteristics, and cell proliferation. Since changes in carbohydrate structure in tumor cells have been proved to be specific for different tumor entities [20], these molecules have been investigated for cancer diagnosis.

CA19-9 is probably the most extensively validated and investigated biomarker regarding diagnostic but also prognostic and surveillance capacity [21]. Therefore, it is the only FDA-approved biomarker for diagnosis and monitoring of PDAC [22]. For the diagnosis of PDAC in symptomatic patients, a systematic meta-analysis determinate a median specificity of 82% and a median sensitivity of 79% from pooled data of 2283 patients [22]. These results have been replicated in more recent studies on the use of CA19-9 in the diagnosis of PDAC [23,24,25]. Nevertheless, several limitations of CA 19-9 have been identified that impair the value of this biomarker. First, approximately 5–10% of the Caucasian population have a strongly reduced production of CA19-9 due to dependence on the Lewis antigen [22]. Therefore, current studies aimed to define different types of CA19-9 secretion in consideration of the patient’s Lewis antigen status and other features [26,27]. Furthermore, there are several distracting conditions in which an elevation of CA19-9 has limited validity, like obstructive jaundice, liver cirrhosis, (chronic) pancreatitis and cholangitis [28]. Hence, the diagnostic value in symptomatic patients, especially considering the differentiation between malignant and benign cause of obstructive jaundice is limited, and require repetitive measurements after relief of jaundice [22]. Additionally, the low positive predictive value of CA19-9 restrains its application as a screening instrument for larger populations [29].

Other carbohydrate antigens have been evaluated regarding their diagnostic value for the early diagnosis of pancreatic cancer. Previous studies have linked CA125 and CA72-4 to PDAC [30] as well as CA50, CA199 and CA242 [31,32,33]. A solitary diagnostic potential could not be verified for any of these carbohydrate antigen biomarkers; however, they might help to discriminate between benign and malignant pancreatic lesions in combination with CA19-9 [30].

Similarly, CEA elevation has lower sensitivity and specificity than CA19-9 in early diagnosis of PDAC [23,34]. Thus, CEA seems to have a greater value as a prognostic marker, especially with regard to advanced PDAC when combined with CA19-9 [35].

### 2.2. Micro-RNA, Circulating Tumor DNA (ct-DNA) and Circulating Tumor Cells (CTCs)

Micro-RNAs are small non-coding RNAs that regulate genetic expression post transcriptionally and have been found to be dysregulated in several cancers [36]. A major advantage of micro-RNA as a diagnostic tool is its deposition in different tissue and body fluids like pancreatic tissue, urine, blood, stool and pancreatic juice. Therefore, various (combined) micro-RNA panels have been investigated for early diagnosis of PDAC [37,38].

In a small cohort of six stage I PDAC patients, three micro-RNAs, miR-143, miR223, and miR30e were significantly higher expressed in the urine compared to healthy controls and to stage II-IV PDAC patients [39]. Analysis of salvia from another seven patients with PDAC compared to patients with IPMN (*n* = 2), pancreatitis (*n* = 4) and healthy controls (*n* = 4) showed that hsa-miR-21, hsa-miR-23a, hsa-miR-23b and miR-29c were significantly upregulated, with a sensitivity of up to 85.6% and a specificity of 100% [40]. In stool samples of 30 PDAC patients, MiR-21 and miR-155 levels were elevated and miR-216 levels decreased compared to 10 patients with chronic pancreatitis, (CP) resulting in a combined sensitivity and specificity of 83.33% [41]. In a case control study including 409 PDAC, 25 CP and 312 healthy participants, two blood-derived micro-RNA panels were established that could better distinguish PDAC patients from healthy controls compared to CA19-9 [42].

Additionally, it has been shown that micro-RNA can be used as a biomarker for precursor lesions of pancreatic cancer [43,44,45,46], which makes it interesting as a potential screening tool in the asymptomatic population.

Another potential diagnostic tool is liquid biopsy in the form of circulating tumor DNA (ct-DNA) or circulating tumor cells (CTCs). The most limiting factor in early stages of PDAC is the low amount of ct-DNA and CTCs restricting the feasibility as biomarkers [47,48]. However, it can be assumed that with increasing sensitivity of ct-DNA and CTC detection techniques, the role of these biomarkers will gain importance. The quantity of circulating ct-DNA in patients with PDAC is significantly higher than in patients with IPMN and healthy controls [49]. Furthermore, the presence of GNAS and KRAS mutation in circulating ct-DNA can help to distinguish patients with premalignant IPMNs from benign pancreatic tumors [49]. A combination biomarker panel including the quantitative amount of ct-DNA, CA19-9 and Thrombospondin-2 could significantly improve the diagnostic power of CA19-9, especially in the detection of potentially curable stage I PDAC patients and the discrimination from CP and IPMN [50]. In addition to the only quantitative measurement, different approaches have tried to identify specific alterations in ct-DNA as a possible tool for non-invasive diagnosis of PDAC. For example, 5-hydroxymethylcytosine signatures or differently methylated markers in ct-DNA of PDAC patients were found compared to healthy controls [51,52].

### 2.3. Metabolomics

During carcinogenesis, specific metabolic changes occur that could be used for early detection of pancreatic cancer. In the case of pancreatic cancer progression, cancer cells undergo metabolic reprogramming including an upregulation of glycolysis and the pentose phosphate pathway as well as alterations in amino acid metabolism which lead to drug resistance and tumor progression [53]. Interestingly, specific metabolic changes can be detected up to 18 months before conventional PDAC diagnosis. Even more up to 30 months before PDAC diagnosis patients developed hyperglycemia, followed by precachexia with a decrease of serum lipids, body weight and abdominal subcutaneous fat [54]. In the urine of 96 early stage PDAC patients a specific six-metabolite panel (trigonelline, glycolate, hippurate, creatine, myoinositol and hydroxyacetone) was upregulated compared to 56 healthy controls [55]. Another case-control study including 914 PDAC patients could reveal an advantage for a nine metabolites panel mainly consisting of complex lipids, fatty acids and related metabolites, compared to CA19-9 in discriminating early stage PDAC from CP [56]. The authors claimed clinical significance of the panel with a negative predictive value of 99.8% that would have allowed to change the diagnostic workup and treatment stratification for one third of the patients included [56].

To discriminate PDAC from distal cholangiocarcinoma can be challenging. A nine-metabolite biomarker panel, consisting of mainly lipids, amino acids and CA19-9 allowed to distinguish PDAC (*n* = 38) form distal cholangiocarcinoma patients (*n* = 34), with a AUC of 0.888 [57].

In conclusion, specific metabolic PDAC signatures are detectable, but larger clinical trials validating these are urgently warranted. To date metabolic biomarkers can support the diagnostic power of CA19-9 especially in discriminating benign from malign pancreatic lesions.

### 2.4. Further Biomarker Approaches

Finally, a plethora of further biomarkers aiming to improve diagnostic accuracy in early-stage PDAC are published to date.

#### 2.4.1. Multimarker Panels

Most biomarkers have been identified as a part of multimarker panels in combination with CA 19-9. As an example, the combination of eight proteins (S100A11, ITGB5, PPY, ERBB3, SCAMP3, RET, 5_NT, CEACAM1) allowed sufficient discrimination between 71 early stage I/II PDAC patients and 72 healthy individuals (AUC 0.85) [58]. Further potential biomarkers that have shown an improvement of diagnostic accuracy in combination with CA19-9 are Thrombospondin-2 [50,59] and Cyfra 21-1 [60]. Especially for the identification of early-stage, potentially resectable PDAC a combined biomarker panel of plasma tissue factor pathway inhibitor (TFPI), tenascin C (TNC-FN III-C), and CA 19-9 levels improved discrimination of early stage PDAC from benign pancreatic lesions [61]. In line, also inflammatory cytokines can improve diagnostic discrimination between benign and malignant pancreatic lesions in patients with obstructive jaundice as shown for a biomarker panel of IL-8, CXCL10 and IL-15 [62].

#### 2.4.2. Alternative Splicing Variants and Methylation

Alternative splice variant proteins are potential biomarkers for early- and advanced stage PDAC and are currently evaluated in a preclinical setting [63]. By using data from the Cancer Genome Atlas, Yu et al. could identify different alternative splice variants that were significantly associated with overall survival in pancreatic cancer, e.g., DAZAP1, RBM4, ESRP1, QKI, and SF1 [64]. Nevertheless, no biomarker for the early diagnosis of pancreatic cancer has been established based on alternative splice variant proteins so far.

A promising approach is the analysis of specific methylation signatures of cfDNA for non-invasive diagnosis of PDAC and its precursor lesions. For example, a high DNA promoter methylation of BNC1 and ADAMTS1 was demonstrated to be specific for serum of PDAC patients with a promising opportunity to be further evaluated as a liquid biopsy biomarker [52]. On the other hand, quantitative DNA methylation levels of specific genes in cfDNA can be used to distinguish PDAC from CP and benign biliary obstruction, for example NPTX2, SPARC, and UCHL1 methylation levels [65]. These changes in DNA methylation in the course of carcinogenesis from (inflammatory) precursor lesions to invasive PDAC have the potential to function as biomarkers for the surveillance of risk groups by periodic analysis of gene-specific methylation state [65].

#### 2.4.3. Exosomes

Exosomes are small extracellular vesicles containing nucleic acids, proteins and lipids secreted by multivesicular bodies through exocytosis [66]. To identify exosomes, specific intravesicular proteins can be used, e.g., synthenin-1 [67]. In a discovery-stage clinical study, Kitagawa et al. investigated the expression of four exosomal mRNA molecules (mRNAs: CCDC88A, ARF6, Vav3, and WASF2) and five exosomal small nucleolar RNA molecules (snoRNAs: SNORA14B, SNORA18, SNORA25, SNORA74A, and SNORD22) from 27 PDAC patients compared to 13 healthy controls, whereby they could achieve a AUC of >0.9 for the diagnosis of early stage PDAC [68]. Another interesting aspect of exosome is the tumor specific expression of exosome surface proteins, which can be used as biomarkers for PDAC. Castillo and colleagues characterized six PDAC-specific exosome surface proteins containing CLDN4, EPCAM, CD151, LGALS3BP, HIST2H2BE, and HIST2H2BF [69]. These proteins were found to be part of the tumorspecific “surfaceome” of PDAC exosomes and were suggested as a biomarker panel for the diagnosis of PDAC by the authors. With circulating levels of Glypican-1 (GPC1) as a specific proteoglycan on cancer exosome surface healthy individuals, patients with benign pancreatic and different stages of pancreatic cancer could be adequately distinguished. Furthermore, levels of GPC1 correlated with tumor burden and with pre- and post-surgical survival [70].

#### 2.4.4. Radiomics

Unlike the previously presented biomarkers, several approaches have tried to combine radiologic imaging technology with specific molecules that improve the detection of PDAC, mostly in a preclinical setting. For example, Bausch et al. have evaluated Plectin-1 as a contrast agent for single photon emission computed tomography in an orthotopic and liver metastasis murine model of PDAC and showed that Plectin-1 expression was upregulated in PDAC tissue [71]. Another approach is the use of established biomarkers for pancreatic cancer tissue as an immunological target for multimodal imaging, mostly as immuno- positron emission tomography (PET) with radiolabeled high-affinity specific antibodies. Lohrmann and colleagues have developed a CA19-9 antibody HuMab-5B1 that showed a high uptake in PDAC tissue, especially regarding the detection of lymph node metastasis by using immuno-PET technology in a phase I clinical trial [72]. To distinguish inflammatory from malignant processes, radiolabeled neurotensin analogues for PET CT imaging have been evaluated in several preclinical studies with promising results for the diagnosis of PDAC [73].

#### 2.4.5. Outlook

More recent approaches make use of multi-omics analysis, integrating comprehensive analysis of genetic, epigenetic, transcriptional alterations and changes in protein expression, metabolites, and microorganisms extracted from large data sets [74]. With increasing insight of carcinogenesis by the analysis of large data sets supported by deep learning algorithms, new biomarkers are developed that pledge a further improvement in the diagnosis of early-stage PDAC [75,76].

## 3. Biomarkers as Predictors for Therapy Response and Targeted Therapy in PDAC

Another field of interest are biomarkers that can be used as predictors for therapy response and to spare toxicity. Several new therapeutic targets have been discovered and linked to individual tumor characteristics which can be considered as biomarkers for targeted therapy. Nevertheless, todays guidelines only recommend an evaluation of therapeutical efficacy with a comparative imaging technique using the RECIST criteria [11].

### 3.1. Prognostic Biomarkers for Cytostatic Chemotherapy

In consideration of the ECOG performance status, different chemotherapy regimens are established in metastatic or locally advanced PDAC [11]. In ECOG 0 to 1 FOLFIRINOX (5-FU, leucovorin, irinotecan, oxaliplatin) and gemcitabine/nab-paclitaxel have shown highest potency regarding survival times [19,77]. For gemcitabine/nab-paclitaxel response a CA19-9 association was shown, with the greatest reduction in the risk of death in patients with CA19-9 levels ≥ 59xULN and in those with a serum level decrease over 90% (median overall survival 13.5 months (CA19-9 decrease >90%), 8.2 months (CA19-9 decrease <90%) [77]. Similarly clear associations with CA19-9 levels were not associated with other substances like the VEGF receptor inhibitor axitinib [78].

For adjuvant therapy, it has been shown that patients with a postoperative CA19-9 level > 90 U/mL did not benefit from adjuvant chemotherapy after surgery [79]. Furthermore, Lee et al. evaluated the feasibility and clinical utility of ctDNA for perioperative chemotherapy in PDAC. In their study, they showed that 83% of patients with preoperative detectable ctDNA recurred after curative-intended resection defining a subgroup that might specifically benefit from neoadjuvant treatment [80]. Postoperative detection of ctDNA was linked to an increased risk of relapse and significant worse overall survival (OS) [80].

For gemcitabine several studies have identified different biomarkers as predictors for therapy response due to its dependence on physiologic nucleosid transporter proteins [81]. Human ENT1 (hENT1) expression has shown to be a prognostic biomarker for response to gemcitabine [82,83,84,85,86]. An analysis from the ESPAC-3 trial cohort revealed in 434 PDAC patients for low tumor hENT1 expression a mOS of 17.1 months compared to 26.2 months for patients with high tumor hENT1 expression [84]. Therefore, evaluating hENT1 expression could be decision relevant before gemcitabine induction [84,87,88]. High levels of deoxycytidine kinase (dCK), which transforms gemcitabine in its active form via phosphorylation, has been associated with improved OS in gemcitabine-treated patients [89]. Vice versa, a high expression of tumor ribonuclotid reductase (RRM1/2) has shown to decrease the effect of gemcitabine [90], and therefore has been identified as a relevant therapeutic target [91]. Other predictive biomarkers are e.g., enzymes that are involved in the metabolization of gemcitabine like cytidine deaminase and deoxycytidylate deaminase [92]. In conclusion, reliable data on the clinical feasibility of these approaches is still poor but biomarkers involved in gemcitabine resistance are a promising target for the development of new targeted therapeutics.

Regarding biomarkers predicting therapy response to nab-paclitaxel, several studies have assumed that the expression of SPARC (secreted protein acid and rich in cysteine) in PDAC stroma plays a role as a predictive biomarker [93]. However, this hypothesis was disproved for the treatment response to nab-paclitaxel [94,95] but high cytoplasmatic SPARC expression was associated with a worse response to gemcitabine [96,97].

For 5-FU therapy, resistance was linked to overexpression of multidrug resistance-associated protein 5 [98] and various metabolic enzymes [99]. Last, dihydropyrimidine dehydrogenase (DPD) has been established as a significant biomarker for 5-FU therapy. Upregulation of DPD [100] and upstream TUG1 [101] have been associated with a worse response to 5-FU based therapy. On the other hand, DPD deficiency has been related to 5-FU toxicity and therefore worse prognosis [102]. For that reason, today it is guideline recommended to test patients for DPD deficiency before using regimens containing 5-FU, Capecitabin or Tegafur [103].

Regarding therapeutical response to irinotecan, high carboxylesterase 2 expression was associated with increased OS in mFOLFIRINOX treatment [104]. Nanoliposomal Irinotecan, seems less effective on pancreatic cancer cells with high circulating levels of IL-8 as a marker for TAK1 activation [105].

For oxaliplatin, it has been shown that an upregulation of the nucleotide excision pathway is crucial for poor response to platin-based chemotherapy [106]. Therefore, molecules involved in nucleotide excision pathways have been identified as potential predictive biomarkers for platin-based chemotherapy, especially ERCC1 [107,108]. Furthermore, recent studies have shown that alterations of DNA mismatch repair genes (see below) in PDAC are a positive predictive marker for a therapy response to a platin-based chemotherapy regimen [109]. Table 1 summarizes current evidence on biomarkers and individual drugs.

### 3.2. Predictive Biomarkers for Targeted Therapy in PDAC

The most commonly mutated gene in PDAC is KRAS, which can be found in >90% of patients [112]. So far only the small subset of KRAS^G12C^ mutated patients can be targeted by FDA-approved sotorasib or adagrasib. In a phase 1 trial, sotorasib achieved in 9 patients with different solid tumors a stable disease in six and a partial response (PR) in one patient [113], with none of those diagnosed with PDAC. Regarding adagrasib, the CRYSTAL-1 phase 2 clinical trial showed promising results in KRAS^G12C^-mutated heavily pretreated solid tumors, including one patient with PDAC with a confirmed partial response [114]. Disappointingly, in PDAC only 3% of patients harbor a KRAS^G12C^ [115].

Biomarkers that gain growing interest are alterations of the DNA damage repair (DDR) including mutations of BRCA1/2, PALB2, ATM/ATR, CHEK2 and RAD51. Several studies proved effectivity of PARP inhibitors e.g., olaparib, talazoparib and rucaparib in patients’ DDR alterations [116,117,118]. In the phase 3 POLO trial maintenance therapy, olaparib significantly prolonged progression-free survival compared to placebo in germline BRCA1/2 mutation carrier (7.4 months vs. 3.8 months) [119]. BRCA testing at least in advanced stage PDAC patients should be a standard of care testing [109].

As in other tumor entities, immune checkpoint inhibitors have also shown encouraging results in PDAC [116,117]. As predictors for immunotherapy, a high tumor mutational burden (TMB), measured in mutation per megabase, high microsatellite instability (MSI-high) and mismatch repair deficiency (dMMR) (enhanced expression of MLH1, MSH2, MSH6 or PMS2) were identified [117]. In a phase 2 trial, pembrolizumab markedly improved PFS and OS in patients with dMMR/MSI-high solid tumors [120]. Therefore, current guidelines recommend routine testing of MSI-high and dMMR in patients with PDAC. Although, again, only about 1% of PDAC patients are eligible [121].

In a phase 3 trial, the EGFR-inhibitor erlotinib added a marginal but significant benefit on PFS and OS combined with gemcitabine compared to only gemcitabine. Interestingly, the EGFR expression on tumors was not determinant, but the clinical appearance of a rash under erlotinib was prognostic [122].

A promising approach in targeted therapy is to address fusion of tropomyosin receptor kinase gene 1, 2 or 3 (NTRK1,2,3), which is present in <1% of PDAC [123]. In case of detected fusion, a TRK inhibitor therapy (e.g., entrectinib, larotrectinib) proofed benefit in two phase 2 trials [118]. The analysis of total 4 PDAC patients with NTRK or SCL4-ROS1 fusion revealed PR after treatment with entrectinib [124] or larotrectinib [125].

Further less established potential biomarkers in PDAC could be ALK rearrangements and their inhibition by e.g., crizotinib, ceritinib, alectinib [126,127], or the targeting of NRG1 fusion in KRAS wildtype patients with a EGFR-targeted therapy (Afatinib, Erlotinib) [128]. In 3 PDAC patients with NRG1 fusion, EGFR-targeted therapy resulted in a PR in two of them [128]. Loss of CDKN2A, present in up to 47% of PDAC patients, can be targeted by CDK4/6 inhibitors (palbociclib, ribociclib, abemaciclib) [118], but today evidence in PDAC relies only on preclinical studies [129].

In conclusion, several biomarkers have been identified as predictors for the effectivity of chemotherapeutical regimens and targeted therapies (see Table 2). However, data quality in PDAC remains mainly poor and their clinical value will have to be explored in future studies. At the present stage, these biomarkers can help to predict therapy response and toxicity of established chemotherapeutical substances and facilitate individual treatment.

## 4. The Role of Biomarkers in Surveillance after Curative Surgery

Finally, to improve postoperative surveillance after curatively-intended surgery with biomarkers could be highly valuable.

Regarding the clinical use of serum biomarkers for postoperative surveillance, the only established molecule is CA19-9 in current clinical use [10]. Despite a moderate specificity of maximum 89% and sensitivity of maximum 89% [131], studies have shown that elevated CA19-9 can precede radiographic detected recurrence up to three to six months [132]. Li and colleagues showed that early detection of CA19-9 as a marker for PDAC recurrence after resection can improve patients’ prognosis regarding disease-free survival by early start of a salvage chemotherapy [133]. The preoperative elevation of CA19-9 > 210 U/mL and postoperative elevation of CA19-9 > 37 U/mL were shown to be independently associated with an early recurrence of PDAC [134].

Like its use in early diagnosis of PDAC, CEA has shown to be inferior to CA19-9 with a sensitivity of 50% and a specificity of 65% in postoperative surveillance of PDAC (102).

Today, there is no sufficient evidence or well-defined test to use cfDNA or CTCs in postoperative surveillance [135,136,137]. A systematic meta-analysis assessed 19 studies with a cumulative 1872 patients regarding cfDNA, CTC and exosomale DNA in diagnosis and surveillance of PDAC with first promising significance levels [138].

In 157 PDAC patients measurement of plasma soluble iC3b, the cleavage product of complement factor C3b, showed recurrence up to 4 months before imaging (AUC 0.85), what could even be further improved combined with CA19-9 (AUC 0.92) [139].

In conclusion, for now the only applicable biomarker for postoperative surveillance of PDAC remains CA19-9. Despite interesting approaches for the improvement of post-surgery monitoring, previous studies have mainly focused on predicting patients‘ prognosis than on developing biomarkers for postoperative surveillance. Moreover, a recent systematic review of Wang et al. showed a devastating summary of current research: Only CRP to albumin ratio, neutrophil-lymphocytic ratio and lactat dehydrogenase showed an association regarding prognosis of PDAC [140]. Other promising approaches that were included in their review lacked sufficient evidence [140].

## 5. Conclusions

Pancreatic ductal adenocarcinoma is a leading cause of tumor death. Due to a lack of sufficient methods for early detection and adequate surveillance after curative surgery, the prognosis remains worse. Therefore, current interest of research is the development of new biomarkers to improve prognosis of PDAC.

Regarding biomarkers for early diagnosis, CA19-9 is still the only approved tumor marker. Nevertheless, cell free DNA, circulating tumor cells, and tumor-specific microRNA are promising approaches to enhance diagnostic accuracy. Furthermore, metabolic changes in PDAC function as a starting point for the development of tumor-metabolic based biomarkers. The near future will probably supply combined biomarker panels that could replace CA19-9.

**Figure 1:**
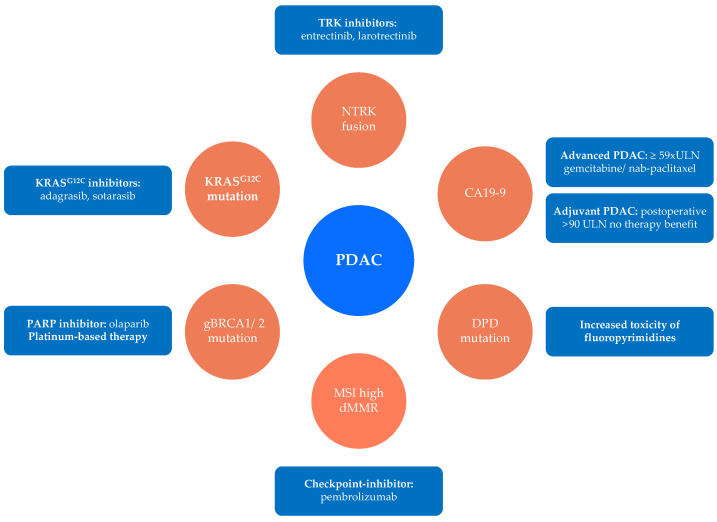
Established biomarkers to direct decision making in pancreatic cancer. Summary of biomarkers which should be considered for therapy decision in PDAC based on sufficient clinical evidence. For advanced PDAC, NTRK, KRAS^G12C^, germline BRCA1/2, and MSI/dMMR should be tested for targeted therapy. To avoid excessive toxicity, DPD-mutation should be evaluated before therapy with a fluoropyrimidine. CA19-9 should be tested regularly before adjuvant therapy and can guide therapy in advanced disease. (gBRCA1/2 = germline BRCA1/2; MSI = microsatellite instability; dMMR = deficient mismatch repair; DPD = dihydropyrimidine dehydrogenase).

Additionally, current therapeutic procedures in metastatic or locally advanced tumor stages are enhanced by the development of predictive biomarkers for response to targeted therapy as well as established chemotherapy regimens. Some of these markers like hENT1 are already near clinical application, while other markers like DPD for toxicity of 5-FU have been implemented, see Figure 1.

In conclusion, biomarkers that should be considered for decision making in pancreatic cancer based on clinical evidence are summarized in Figure 1. BRCA1/2 germline testing has shown to predict therapy response to platin-based chemotherapy based on sufficient clinical evidence and can be considered for targeted maintenance therapy with PARP inhibitors. Before using a fluoropyrimidine-based chemotherapy regimen, DPD testing should be performed to avoid severe toxicity. For targeted therapy in advanced stage PDAC, NTRK fusion, KRAS^G12C^ mutation and MSI/dMMR testing should be routinely performed. Finally, CA19-9 can predict therapy response to a gemcitabine/nab-paclitaxel chemotherapy regimen as well as adjuvant chemotherapy after surgery.

Last, the development of biomarkers to predict early recurrence of PDAC after resection is still lacking. Despite the investigation of prognostic biomarkers to identify high-risk patients for early recurrence, no biomarker but CA19-9 is used for monitoring after surgery.

To conclude, the progress in the understanding of pathogenetic changes in PDAC and the improvement of genetic diagnostics are assumed to supply precise non-invasive biomarkers for PDAC in the near future.

## Figures and Tables

**Table 1:** Biomarkers as predictors for therapy response to specific cytostatic drugs.

Drug	Biomarker	Source	Prognosis	Evidence	Ref.
Gemcitabine	hENT1-high	Tumor	↑	IB	[84]
	Exosomal miRNA-106b	Blood	↓	Preclinical	[110]
	Deoxycytidine kinase	Tumor	↓	Preclinical	[89]
	SPARC	Tumor	↓	Preclinical	[96]
Nab-Paclitaxel	CA19-9	Blood	↓	IB	[77]
5-FU	Multidrug resistance associated protein 5	Tumor	↓	Preclinical	[98]
	Dihydropyrimidine dehydrogenase	Tumor	↓	IB	[100]
Irinotecan	Carboxylesterase 2	Tumor	↑	III	[104]
Nal-Irinotecan	IL8	Blood	↓	Preclinical	[105]
Oxaliplatin	ERCC1	Tumor	↓	III	[107]
	BRCA1/2, PALB2	Serum	↑	III	[111]

**Table 2:** Biomarkers as predictors for therapy response to targeted therapies.

Drug	Target	Biomarker	Frequency	Evidence	Ref.
Pembrolizumab	PD-1	MSI-high, dMMR, TMB-high	<1%	IIa	[120]
Olaparib	PARP	BRCA1/2, PALB2, ATM/ATR, CHEK2, RAD51 mutation	14–24%	Ib	[119]
Larotrectinib Entrectinib	TRK	NTRK-fusion	<1%	IIa	[125]
Ceritinib Crizotinib Alecitinib	ALK	ALK rearrangement	<1%	IV	[126]
Afatinib Erlotinib	EGFR	NRG1 fusion	<1%	IV/III	[128]
Trametinib	BRAF	BRAF deletion	1%	IV	[130]
Sotorasib	KRAS G12C	KRAS G12C mutation	1%	IV	[113]
Palbociclib Ribociclib Abemaciclib	CDK4/6	Loss of CDKN2a	47%	IV	[129]
